# Genetic polymorphism and evolutionary differentiation of Eastern Chinese Han: a comprehensive and comparative analysis on KIRs

**DOI:** 10.1038/srep42486

**Published:** 2017-02-16

**Authors:** Caiyong Yin, Li Hu, Huijie Huang, Yanfang Yu, Zheng Li, Qiang Ji, Xiaochao Kong, Zhongqun Wang, Jinchuan Yan, Jiangwei Yan, Bofeng Zhu, Feng Chen

**Affiliations:** 1 Department of Forensic Medicine, Nanjing Medical University, Nanjing, Jiangsu, 210029, China; 2 Department of Cardiology, Affiliated Hospital of Jiangsu University, Zhenjiang, Jiangsu 212001, China; 3 CAS Key Laboratory of Genome Sciences and Information, Beijing Institute of Genomics, Chinese Academy of Sciences, Beijing, China; 4 Department of Forensic Genetics, School of Forensic Medicine, Southern Medical University, Guangzhou 510515, P. R. China; 5 Key Laboratory of Shaanxi Province for Craniofacial Precision Medicine Research, College of Stomatology, Xi’an Jiaotong University, Xi’an, Shaanxi 710004, P. R. China; 6 Clinical Research Center of Shaanxi Province for Dental and Maxillofacial Diseases, College of Stomatology, Xi’an Jiaotong University, Xi’an, Shaanxi 710004, P. R. China

## Abstract

Killer cell immunoglobulin-like receptor genes, namely KIRs, cluster together within the 160 kb genomic DNA region. In this study, we used PCR-SSP approach and successfully identified the genotype of 17 KIR genes in 123 independent healthy donors residing in the Jiangsu province, China. All individuals were positive at the 7 genes. The observed carrier gene frequencies (OFs) of remaining 10 KIRs ranged from 14.63% (KIR2DS3) to 95.93% (KIR3DL1). We found 27 distinct genotypes excluding KIR1D. The most frequent occurred in 63 individuals (51.22%). The linkage disequilibrium analysis signified 29 positive and 6 negative relations in 45 pairwise comparisons. To study population differentiation, we drew a Heatmap based on the data of KIRs from 59 populations and conducted Hierarchical Clustering by Euclidean distances. We next validated our results by estimating pairwise D_A_ distances and illustrating a Neighbor-Joining tree, as well as a MDS plot covering 3 additional Chinese Han groups. The phylogenetic reconstruction and cluster analysis strongly indicated a genetically close relationship between Eastern and Jilin Hans. In conclusion, the present study provided a meritorious resource of KIR genotyping for population genetics, and could be helpful to uncover the genetic mechanism of KIRs in immune disease in the future.

Natural killer (NK) cells, associated with the innate immune response, are considered as the first line of defense against both infected and malignantly transformed cells^1^. As a kind of bone marrow-derived lymphocyte, NK cells use specific cell surface receptors to distinguish healthy cells and diseased cells^2^. Like T cells, NK cells possess the qualified characteristics of the adaptive immune system, including the production of memory cells that persist following antigen invasion and the ability to create a secondary recall response^3^. Whether NK cells produce activation or inhibitory function depends on the varieties of their surface receptors. NK cells express three main families of receptors: (i) the highly polymorphic killer cell immunoglobulin-like receptors (KIRs) which specifically interact with classical MHC Class I molecules, (ii) the non-polymorphic C-type lectin (CD94/NKGs) receptors which bind to the non-classical MHC molecule HLA-E, and (iii) the immunoglobulin-like transcripts^4^. Killer cell immunoglobulin-like receptors (KIRs), defined as specific cell surface receptors, are a group of glycoproteins expressed on the surface of both NK cells and a few subsets of T cells. Upon the interaction with polymorphic human leukocyte antigen (HLA) class I molecules on the surface of target cells and other ligands, the KIRs participate in various immune responses to different infectious agents. Because of the high diversity of KIR genes, it is reasonable to hypothesize that the polymorphism of the KIRs in combination with HLA genes might affect predisposition to autoimmune disease. Recent genetic experiments demonstrated the associations between KIR and HLA genes with susceptibility to autoimmune diseases including Systemic Lupus Erythematosus (SLE), rheumatoid arthritis, systemic sclerosis and multiple sclerosis^5^,^6^,^7^,^8^,^9^,^10^. NK cells derived from human pulmonary artery hypertension (PAH) patients exhibited a lower level of the KIRs 2DL1/S1 and 3DL1 expression as well as a great disruption of 3DL1 receptor associated cytolytic function, suggesting a novel and substantive role for KIRs in the occurrence and development of immune associated vascular disease^11^.

The KIRs are encoded by a family of highly polymorphic genes clustered within the leukocyte receptor complex region on human chromosome 19q13.4. All of the KIR genes are encoded within a range of 160 kb genomic sequence, and they cluster together with a genetic distance shorter than 3 kb. As well, the KIR clusters have shown a good deal of gene duplications and unequal crossing over, which lead to a wide range of KIR gene combinations. KIR genes contain a tandem array of highly homologous genes, the number and type of which have the ability to show high diversity in different NK clone cells and individuals^12^. KIR gene nomenclature as defined by the World Health Organization (WHO) subcommittee is based on the structure of the encoded molecules. Accordingly, the number of extracellular immunoglobulin domain (D) could be double (2D) or triple (3D) and therefore the length of the intracytoplasmic tail would be short (S) or long (L). In terms of the order of KIR genes and the gene contents of 15 loci, KIR genes are divided into two haplotypes, A and B. The A haplotype contains at least six encoding inhibitory receptors (KIR3DL3, 2DL3, 2DL1, 2DL4, 3DL1 and 3DL2), one pseudogene (KIR3DP1) and one activating receptor gene (KIR2DS4)^13^. The haplotype B consists of a great variety of subtypes that differ from each other in the combination of stimulatory receptors (KIR2DS1, 2DS2, 2DS3, 2DS5, 3DS1, 2DL2 and 2DL5)^14^. On condition that the presence of KIR2DS1, 2DS2, 2DS3, 2DS5, 3DS1, 2DL2 and 2DL5 are observed, the genotype would be determined as including B. If all of the above-mentioned genes are absent, the genotype would be defined as AA. When one or more of KIR genes belonging to A group are absent, the genotype would be taken as BB. Otherwise, all the remaining genotypes are defined to be AB. Seven KIR genes belonging to B group are located centromeric or telomeric half in B cluster, B group is classified into 2 half groups: C4 half (KIR2DS2, 2DL2, 2DS3 and 2DL5) and T4 half (KIR3DS1, 2DL5, 2DS1 and 2DS5). Herein, the B group is divided into 4 subgroups: C4T4, C4Tx, CxT4, and CxTx. Seven KIRs (KIR2DL1, 2DL2, 2DL3, 2DL5, 3DL1, 3DL2 and 3DL3) have inhibitory functions while five KIRs (KIR2DS1, 2DS2, 2DS3, 2DS5 and 3DS1) exhibit active functions. KIR2DL4 has both inhibitory and active functions^12^. Among all KIR genes, four genes KIR2DL4, KIR3DL2, KIR3DL3 and KIR3DP1 are described as framework genes, and they are present in nearly all individuals^15^.

In the previous research, studies have shown the KIR gene diversity in different geographical populations^16^,^17^,^18^,^19^,^20^,^21^,^22^,^23^,^24^,^25^,^26^. However, more studies are needed to determine the KIR diversity in populations from different geographical areas and to explain the heterogeneity of KIR distributions in various Han populations in China. In our study, we successfully identified the genotype of 17 KIR genes including KIR2DL1, 2DL2, 2DL3, 2DL4, 2DL5, 3DL1, 3DL2, 3DL3, 2DS1, 2DS2, 2DS3, 2DS4, 2DS5, 3DS1 (in the full-length form), 1D (in the deleted form), and two pseudogenes 3DP1 (putative protein product) and 2DP1(no protein expression) by using PCR-SSP method and analyzed the distributions of 17 KIR genes in the Eastern Han population of China. Moreover, we conducted a comprehensive genetic analysis of 62 populations with existing KIR genotyping files using a variety of different analysis strategies, including Heatmap, Neighbor-Joining tree, Multidimensional Scaling plot and so on.

## Methods

### Study samples

Blood samples were collected from a total of 123 randomly selected healthy donors of Han ancestry living in Nanjing, Jiangsu province of China, membership of Eastern China. All participants provided their written informed consent and completed a basic health screen. Also, each participant was interviewed to ensure that no individuals have common ancestry going back at least three generations and their three generations are native of eastern coastal area of China. The whole-blood samples anti-coagulated with ethylene diamine tetraacetic acid (EDTA) were frozen at −80 °C until use. The study was conducted in accordance with the human and ethical research principles of Nanjing Medical University and approved by the ethics committee of Nanjing Medical University.

### DNA isolation

According to the manufacturer’s instructions, we extracted genomic DNA from 300 μl peripheral blood containing ethylene diamine tetraacetic acid (EDTA), using TIANamp Genomic DNA Kit (TIANGEN, Beijing, China). The quality and quantity of extracted DNA samples were determined by NanoDrop 2000 (Thermo Fisher Scientific, Waltham, USA). The optimal density values used to evaluate the concentration and purity of extracted DNA reflected by the A260/280 values (1.7 to 1.9). The concentration was adjusted to 20–40 ng/μl.

### KIR PCR-SSP genotyping

KIR genes were genotyped for the presence or absence of the 17 KIR genes, including KIR2DL1, 2DL2, 2DL3, 2DL4, 2DL5, 3DL1, 3DL2, 3DL3, 2DS1, 2DS2, 2DS3, 2DS4, 2DS5, 3DS1 (in the full-length form), 1D (in the deleted form) and two pseudogenes, 3DP1(putative protein product) and 2DP1(no protein expression) using PCR-SSP method with a commercially available KIR GENOTYPING SSP KIT (Invitrogen, California, USA). The PCR amplification was performed with the PCR system in a reaction mixture volume of 10 μl consisting of 4 μl pre-aliquoted PCR buffer, 0.06 μl Taq polymerase, and 30 to 50 ng of template DNA. Temperature cycling conditions for PCR reactions were as follows: denaturation for 1 minute at 95 °C, followed by 30 cycles for 20 seconds at 94 °C, 20 seconds at 63 °C, 1.5 minutes at 72 °C, a elongation step for 10 minutes at 72 °C and finally hold in 4 °C. PCR products were visualized under ultraviolet light after electrophoresis in 1.5% agarose gel well mixed with 10% v/v ethidium bromide. Negative controls were performed for each gene while positive internal controls were performed for each lane. False reactions that yielded no internal control bands were repeated.

### Statistical analysis

The observed carrier frequencies (OFs) of the KIR genes were determined by the number of positive typing reactions. Based on the assumption of Hardy-Weinberg equilibrium, we calculated the estimated gene frequencies (GFs) using the formula, GF = 1−(1−OF)^1/2^. The GF is determined by the OF of the KIR gene in all individuals. Package “pheatmap” (https://cran.r-project.org/web/packages/pheatmap/index.html) based on statistical software R version 3.2.5 (https://www.r-project.org/) was used to draw a Heatmap containing Eastern Chinese Han and 58 other populations with complete KIR genotyping files of 16 KIR genes exclusive of KIR1D which are Jilin Han^27^, Shaanxi Han^18^, Shenzhen Han^28^, Sichuan Han^29^, Xinjiang Han^30^, Yunnan Han^22^ and the complete list of 52 populations in HGDP-CEPH^31^,^32^ distributed around the world. The Heatmap is constructed using Hierarchical Clustering algorithm based on Euclidean distance. The Hierarchical Clustering model generally refers to the assumption that irreducible correlation functions are described by the hierarchical relations: ξ_n_ = Q_n_ξ^n−1^_2_, where ξ_n_ is the nth order correlation function, and the Q_n_ is constant^33^,^34^. The D statistic included in recognized “Genetics” package (https://cran.r-project.org/web/packages/genetics/index.html) was used to conduct linkage disequilibrium (LD) analysis (KIRs whose OFs = 100% were excluded). The calculated formula and according statistics principle reveal the sign of coefficient D which represents the same or opposite allelic association^35^,^36^. Specifically to KIR genes, the completely positive LD (D = 1) indicates both loci are present or absent simultaneously. Oppositely, the complete negativity (D = −1) means just only one of the two loci is present. According to the observed carrier frequencies data of 13 variable KIR genes (KIR-2DL1, 2DL2, 2DL3, 2DL4, 2DL5, 3DL1, 3DL2, 3DL3, 2DS1, 2DS2, 2DS3, 2DS5 and 3DS1) from the above-mentioned 59 populations and 3 other Han populations including Shanghai Han^25^, Hong Kong Han and Singapore Han^19^, Dispan software^37^,^38^ was utilized to generate the D_A_ genetic distances^38^,^39^ and relevant significances without correction. According to the estimation formula, D_A_ is a direct calculation of genetic association between any 2 populations^38^ whilst F_ST_ is a relative measure of genetic differentiation given the total genetic variation presents in the population^40^. From an accuracy point of view, D_A_ genetic distance was commonly employed in studying KIRs^19^,^41^,^42^ because Nei D_A_ distance is proved to possess the highest probability of obtaining the correct branching pattern of a phylogenetic tree^43^. By using the distance matrix, we drew a Neighbor-Joining tree and assessed its reliability by interior branch test using Mega version 6.0^44^. As for Neighbor-Joining algorithm, it’s a simplified version of the Minimum Evolution (ME) method, which doesn’t require the assumption of a constant rate of evolution mentioned in Hierarchical Clustering algorithm. The N-J tree reconstruction starts with a starlike tree with no hierarchical structure and the necessary assumption is that there is no clustering of OTUs (operational taxonomic units)^45^. To validate the genetic relationship of the studied populations, we illustrated a Multidimensional Scaling (MDS) plot using the “MASS” packages (http://www.r-tutor.com/category/r-packages/mass). Chi-square test was conducted by SPPS 22.0 to depict the distribution variances between Eastern Chinese Han and Jilin Han (Northeast China)^27^, Shaanxi Han^18^, Xinjiang Han (Northwest China)^30^, Yunnan Han^22^, Sichuan Han (Southwest China)^29^, Shenzhen Han^28^, Hong Kong Han (Southeast China), Singapore Han (overseas Chinese)^19^, and Shanghai Han^25^.

## Results and Discussion

### Observed KIR carrier frequencies

Table 1 lists the distribution of the OFs of 17 KIR genes in our studied Eastern Chinese Han population. The data showed that 4 framework KIR genes including KIR2DL4, KIR3DL2, KIR3DL3 and KIR3DP1 were observed in all individuals. Additionally, KIR2DL1, KIR2DL3 and KIR2DP1 had the highest OF (100.00%) followed by KIR3DL1 (95.93%) while KIR2DS3 had the lowest OF (14.63%). Accordingly, the calculated GFs ranged from 7.60% to 100.00%.

### KIR genotypes, haplotypes and Linkage group

**Figure 1:**
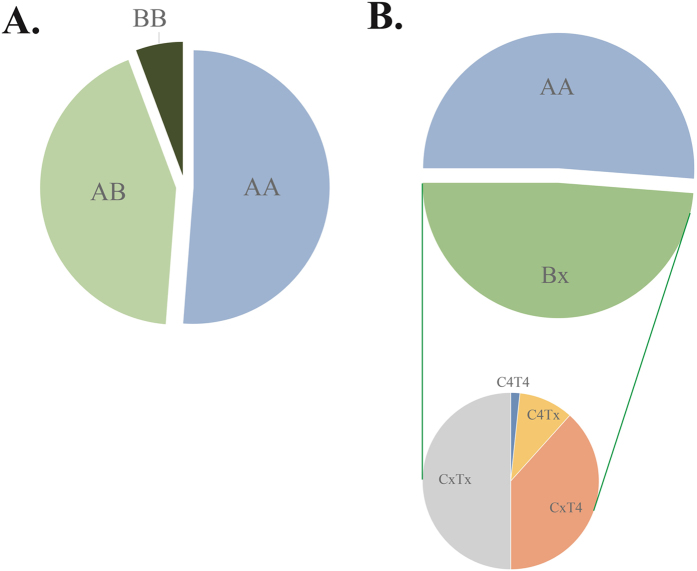
The classification and relative proportions of haplotypes, genotypes and linkage groups based on the genotyping profiles observed on 16 Killer cell immunoglobulin-like receptors (KIRs) in the Eastern Han population (n = 123).

The genotypic profiles of 123 Eastern Chinese Han individuals were summarized in Table 2. The black and white box represented the presence and absence of 17 KIR genes in Eastern Chinese Han population. The haplogroup information was obtained from the website (http://www.allelefrequencies.net/kir6001a.asp). Next, we defined the genotypes and linkage groups. With the exclusion of KIR 1D, we detected a total of 27 distinct genotypes. After careful comparison of the identified genotypes in the database, we didn’t observe any new genotypes. The most common genotype (KIR3DL1-2DL1-2DL3-2DS4-2DL4-3DL2-3DL3-2DP1-3DP1, n = 63, ratio = 51.22%) was the same to Xinjiang Han, Yunnan Han, Sichuan Han and Shenzhen Han, among which 32 individuals carried KIR1D (Table 2). In Fig. 1, the classification of haplotypes, genotypes and linkage groups were shown intuitively. As we can see in Fig. 1A, 7 BB genotypes (5.69%), 53 AB genotypes (43.09%) and 63 AA genotypes (51.22%) were determined. Previous studies have demonstrated a great diversity of KIR genes among different populations from Asia, in which AB accounted for the most prevalent genotype in Shaanxi Han (48%)^18^, Chinese Kazakh (52.8%), and Chinese Uyghur (56.1%)^46^. It revealed a great diversity of KIR gene distribution among different groups. As for linkage group (Fig. 1B), among 60 samples categorized as Bx haplogroup, 30 were from CxTx (50.00%), 23 from CxT4 (38.33%), 6 from C4Tx (10.00%) and 1 from C4T4 (1.67%). Our data provided a clear KIR gene genotype distribution in Eastern Chinese Han population.

### Linkage disequilibrium (LD) analysis

**Figure 2:**
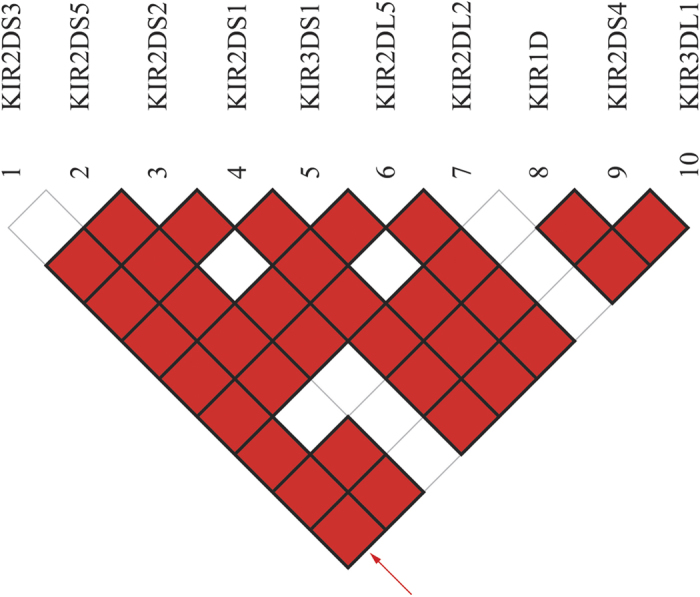
The LD analysis among 10 KIR genes. As shown by the red arrow, the comparisons of the locus KIR2DS3 and the rest 9 loci were arranged in left bottom (9 square grids). The other comparisons were listed as well. The red area encircled by thick black line represented strong linkage relationship.

Since KIR genes are close to each other by approximately 3 kb genomic DNA region, linkage disequilibrium should be taken into consideration, especially for those two nearest-neighbor KIRs (present or absent simultaneously). Therefore, we conducted the LD analysis, in which D was the parameter representing the test statistics linkage disequilibrium coefficient. P-value < 0.05 signified a strong genetic relation between two KIRs. Based on the genotyping profiles of 123 Eastern Chinese Hans, Table 3 and Fig. 2 listed the D coefficients and conspicuous differences of 45 pairs among 10 KIR genes except for 7 KIR genes which appeared in all individuals. The Linkage Disequilibrium comparisons were listed in an inverted triangle in Fig. 2.

The results of LD analysis showed 35 strong relations in total, among which 6 were negative, all associated with KIR1D (1D-3DL1, 1D-2DS4, 1D-2DL5, 1D-3DS1, 1D-2DS1, and 1D-2DS3). The rest 29 relations were significantly positive on different levels. In terms of group classification, 1 relation was from A group (3DL1-2DS4) while 10 pairs linked A and B groups (3DL1-2DL5, 3DL1-3DS1, 3DL1-2DS1, 3DL1-2DS5, 3DL1-2DS3, 2DS4-2DL5, 2DS4-3DS1, 2DS4-2DS1, 2DS4-2DS5, and 2DS4-2DS3) and 18 linkage relations existed within B group. After further demarcation, 6 linkage relations (2DL5-2DL2, 2DS2-2DL2, 2DS3-2DL2, 2DS2-2DL5, 2DS3-2DL5, and 2DS2-2DS3) and 5 (3DS1-2DL5, 2DS1-2DL5, 2DS5-2DL5, 3DS1-2DS5, and 2DS1-2DS5) were found within C4 and T4 subgroups from B family respectively, consistent to the genomic location of the genes. As for 1D, a known 2DS4 variant, we illustrated a very close linkage relationship between 1D and KIR2DS4 which showed the linkage coefficient was −0.0245 (p < 0.001). Thus, the results supported that KIR1D is the variant of KIR2DS4^47^. These findings are consistent to other published studies^48^,^49^,^50^,^51^.

### Evolution significance of KIR cluster and Phylogenetic restructure

In order to investigate the potential role of KIRs in population genetics, we compared the KIR frequencies of Eastern Han and 58 other populations and performed population differentiation analysis. Because KIR1D was excluded from the database, we utilized the rest 16 genes for the Heatmap analysis. The 59 selected populations are from East Europe, West Europe, Africa, North America, Latin America, Asia and Pacific Ocean, which are almost on behalf of human populations all over the earth.

**Figure 3:**
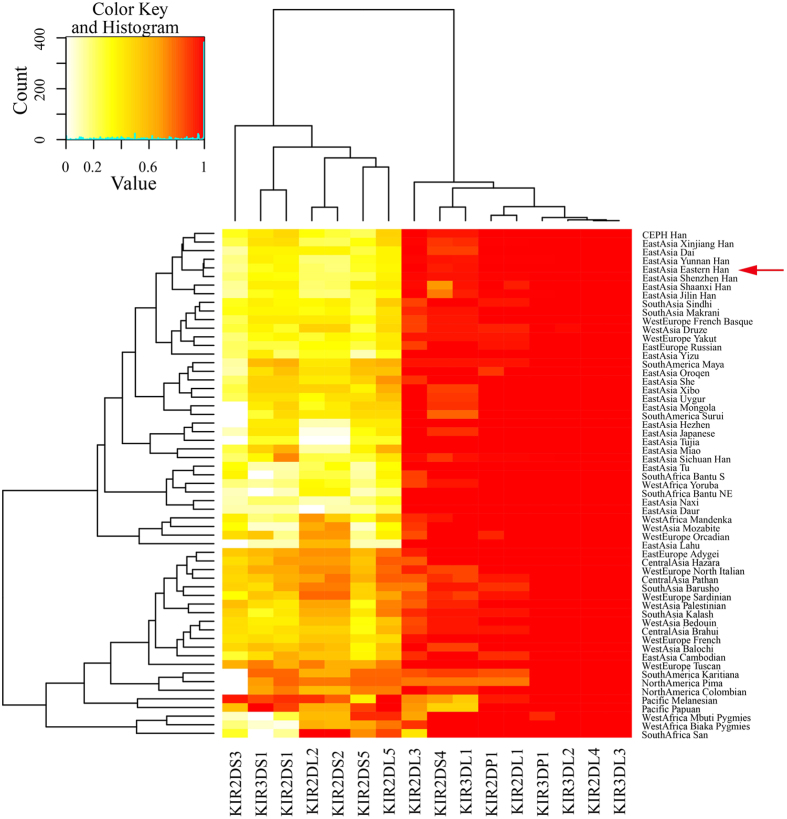
A Heatmap illustrated containing Eastern Han and 58 other populations distributed worldwide, as well as 16 KIR loci in molecular evolutionary structure. The deeper color indicated higher observed carrier frequency from 0 to 1.

In Fig. 3, the deeper color represented higher observed frequency. We found all KIR genes are present in Eastern Han population. However, KIR3DS1 was not detected in African populations such as Bantu NE, Bantu S, Mbuti Pygmies and San groups. Similarly, 2DS3 was missing in some populations from East Asia (Hezhen, Lahu, Mongola, and Tujia) and America (Karitiana, Pima, Surui, and Colombian). With the knowledge that EDAR variant emerged with ameliorative phenotypes in East Asian, especially Central Chinese by natural selection^52^, we speculated that KIR3DS1 was beginning to surface and KIR2DS3 disappeared gradually due to evolutionary pressure such as microbial infection, communicable disease or natural environment on the route of “out of Africa”. However, the potential causes remained to be discovered. The Hierarchical Clustering method was employed to reconstruct the cluster trees and molecular evolutionary structures based on Euclidean distance. The populations and KIRs were listed corresponding to the population genetic and homologue analysis, respectively (Fig. 3). Firstly, we found that molecular evolutionary structure was divided into two clusters. The left was composed of KIR genes defined as B group, while the right consisted of A group and KIR2DP1. The results were consistent with the deduced classification clones from original research^14^. Interestingly, KIR2DS1 clustered with KIR3DS1, KIR2DL2 and 2DS2 were from the same branch, matching up with the classification of linkage subgroups (C4, T4). Secondly, from the phylogenetic structure, two main clusters existed according to the relative OFs of B group loci. The lower cluster differed from the top by higher OFs for most B haplotype KIRs (especially the lowest 5 populations from Oceania and Africa including Melanesian, Papuan, Mbuti Pygmies, Biaka Pygmies and San). The studied Eastern Han labelled with red arrow resided in the top cluster. Obviously, Han populations from HGDP-CEPH database including Xinjiang Han, Chinese Dai, Yunnan Han, Eastern Chinese Han, Shenzhen Han, Shaanxi Han, and Jilin Han gathered together in one sub-clade. Moreover, all East Asian populations clustered together in the top right. By utilizing the 16 KIR genes as genetic marker series, the phylogenetic reconstruction analysis confirmed that Eastern Han was close to other Han populations and East Asian populations. The Hierarchical Clustering model provided a preliminary observation of the relations among Eastern Chinese Han and 58 other populations with complete KIR genotyping files of 16 KIR genes from a mathematics angle.

### Phylogenetic structure and MDS analysis

**Figure 4:**
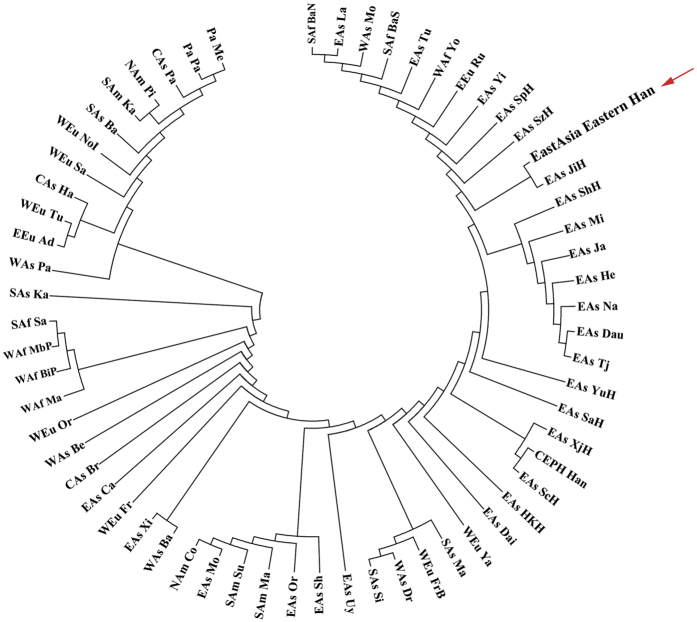
A Neighbor-Joining (NJ) tree composed of Eastern Chinese Han, 52 populations in HGDP-CEPH and 9 reported Han groups (Shanghai Han, Jilin Han, Xinjiang Han, Yunnan Han, Sichuan Han, Shenzhen Han, Shaanxi Han, Hong Kong Han and Singapore Han) was reconstructed using 13 KIR loci. The name abbreviation and order of selected populations are listed as follows. 01-EastAsia Eastern Han, 02-SAf BaN: SouthAfrica Bantu NE, 03-SAf BaS: SouthAfrica Bantu S, 04-WAf BiP: WestAfrica Biaka Pygmies, 05-WAf Ma: WestAfrica Mandenka, 06-WAf MbP: WestAfrica Mbuti Pygmies, 07-SAf Sa: SouthAfrica San, 08-WAf Yo: WestAfrica Yoruba, 09-WAs Mo: WestAsia Mozabite, 10-WAs Be: WestAsia Bedouin, 11-WAs Pa: WestAsia Palestinian, 12-WAs Dr: WestAsia Druze, 13-EEu Ad: EastEurope Adygei, 14-WEu Fr: WestEurope French, 15-WEu FrB: WestEurope French Basque, 16-WEu NoI: WestEurope North Italian, 17-WEu Or: WestEurope Orcadian, 18-EEu Ru: EastEurope Russian, 19-WEu Sa: WestEurope Sardinian, 20-WEu Tu: WestEurope Tuscan, 21-CAs Pa: CentralAsia Pathan, 22-SAs Ma: SouthAsia Makrani, 23-SAs Ka: SouthAsia Kalash, 24-CAs Ha: CentralAsia Hazara, 25-WAs Ba: WestAsia Balochi, 26-SAs Ba: SouthAsia Barusho, 27-CAs Br: CentralAsia Brahui, 28-SAs Si: SouthAsia Sindhi, 29-EAs Uy: EastAsia Uygur, 30-EAs Ca: EastAsia Cambodian, 31-EAs Dai: EastAsia Dai, 32-EAs Dau: EastAsia Daur, 33-CEPH Han, 34-EAs He: EastAsia Hezhen, 35-EAs Ja: EastAsia Japanese, 36-EAs La: EastAsia Lahu, 37-EAs Mi: EastAsia Miao, 38-EAs Mo: EastAsia Mongola, 39-EAs Na: EastAsia Naxi, 40-EAs Or: EastAsia Oroqen, 41-EAs Sh: EastAsia She, 42-EAs Tu: EastAsia Tu, 43-EAs Tj: EastAsia Tujia, 44-EAs Xi: EastAsia Xibo, 45-WEu Ya: WestEurope Yakut, 46-EAs Yi: EastAsia Yizu, 47-Pa Pa: Pacific Papuan, 48-Pa Me: Pacific Melanesian, 49-SAm Ka: SouthAmerica Karitiana, 50-SAm Ma: SouthAmerica Maya, 51-NAm Pi: NorthAmerica Pima, 52-SAm Su: SouthAmerica Surui, 53-NAm Co: NorthAmerica Colombian, 54-EAs ShH: EastAsia Shanghai Han, 55-EAs JiH: EastAsia Jilin Han, 56-EAs XiH: EastAsia Xinjiang Han, 57-EAs YuH: EastAsia Yunnan Han, 58-EAs SiH: EastAsia Sichuan Han, 59-EAs SzH: EastAsia Shenzhen Han, 60-EAs SaH: EastAsia Shaanxi Han, 61-EAs HKH: EastAsia Hong Kong Han, 62-EAs SpH: EastAsia Singapore Han.

In the validation study, we constructed another Neighbor-Joining tree (Fig. 4) by utilizing the observed frequency data of the Eastern Han, 52 populations from HGDP-CEPH, and reported 9 Han groups at 13 KIR loci (KIR2DL1, 2DL2, 2DL3, 2DL4, 2DL5, 3DL1, 3DL2, 3DL3, 2DS1, 2DS2, 2DS3, 2DS5 and 3DS1) with the purpose of revealing genetic relationships among these 62 populations. The results of interior branch test were shown in Figure S1 with the sum of branch length = 0.41784153. The values of D_A_ and significant level were listed in Supplementary Table S1. Without correction, the p-value greater than 0.05 appeared 330 times and the rest 1,561 comparisons were found to be significantly different. The number of significances ranged from 13 in Pacific Melanesian to 60 in Bedouin, Balochi, Brahui, Xibo and Cambodian from Asia. As for Eastern Han, 53 significant differences existed, which is similar to various Han populations, for example 50 for Jilin Han, 53 for Shanghai Han, 54 for Shenzhen Han, Yunnan Han, and Sichuan Han, 55 for Singapore Han, and 56 for Shaanxi Han, and Xinjiang Han. We next shorten the population list to clearly depict the D_A_ distances and according significant differences in Table 4. We found that the Eastern Han had the closest distance with Jilin Han (D_A_ = 0.0006), followed by Yunnan Han (D_A_ = 0.0011), with the farthest distance with Pacific Melanesian (D_A_ = 0.1567).

In the phylogenetic tree, we could clearly find two main clusters. The smaller cluster consisted of Palestinian, Adygei, Tuscan, Hazara, Sardinian, North Italian, Barusho, Karitiana, Pima, Pathan, Papuan and Melanesian. Eastern Chinese Han gathered with Jilin Han in another cluster, following the sub branch containing Shanghai Han, Miao, Japanese, Hezhen, Naxi, Daur and Tujia, then with Yunnan Han and Shaanxi Han. Interestingly, all 7 African populations were genetically closer to populations from East Asia than West Europe, America and Pacific Ocean indicating that Negroid race had a closer relationship to Mongoloid than Caucasian. This finding supported the theory of “Out of Africa”^53^,^54^,^55^,^56^.

Using KIR as the panel of genetic marker, the results showed that Eastern Han from Jiangsu province had the closest relationship with Jilin Han, followed by Shanghai Han, indicating that Eastern Han shared a common ancestry, which was consistent to the findings based on other genetic markers, such as autosomal STRs and Y-STRs by Yao *et al*.^57^. The notable association of Eastern Han with Jilin Han was confirmed by the famous large-scale migration literally called “Crashing into Guandong”. Since A.D. 1,650, to control the rapid expansion of Chinese Han population, Han from Eastern China was promoted by the national policy to migrate to Northeastern China. As a result, Chinese Han accounted for 80% of the total northeastern Chinese during the 18th century. Besides, that Eastern Han was genetically close to Shanghai Han could be explained by the close geographic location and long-term cultural interaction^58^. We also found Hong Kong Han displayed the farthest genetic distance to Eastern Han compared to other Han populations, obviously, which could be explained by the fact that Hong Kong Han mixed with lineage from the United Kingdom^59^,^60^. The genetic characteristics of Eastern Han revealed that subpopulations sharing the same ancestry may have genetically closer relationships than geographically close groups. Wen *et al*. revealed the southward expansion of Han on the basis of variance of Y-chromosomal and mitochondrial DNA^61^. Chen *et al*. analyzed genome-wide SNP variation and demonstrated the population stratification of Hans from 10 provinces including over 6,000 Han Chinese samples^62^. Both studies indicated the north-south migration of Han Chinese. In this research, the relationships between Eastern Han populations and others worldwide were emphasized. The strongest association between Eastern Han and Jilin Han showed the impact of historical event “Crashing into Guandong”, which was a south to north migration route in the history, on genome polymorphisms. Our research utilized KIR as markers to provide new evidences for illustrating the potential Han dispersal. The Neighbor-Joining algorithm optimized the genetic relationships using bioinformatics. The combination of these two entirely different algorithms (Hierarchical Clustering and Neighbor-Joining) provided strong evolutionary evidences of Eastern Han and other various Han groups (Jilin Han, Shanghai Han, and Shaanxi Han).

**Figure 5:**
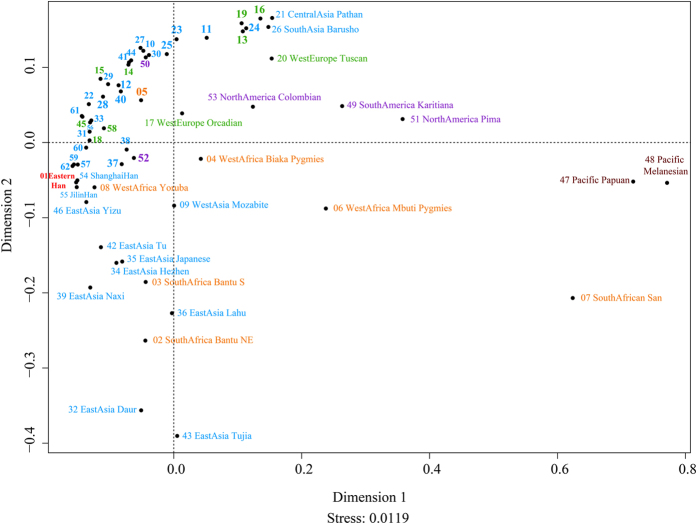
A MDS plot was drew based on KIRs contents in Eastern Han and the above-mentioned 61 populations (Stress = 0.0119). Eastern Han was colored in red and other Asian populations were in blue, African populations were in orange, European populations were in green, American populations were in violet, and Pacific populations were in brown.

Based on the D_A_ matrix achieved from the OFs of 13 KIR genes (KIR2DL1, 2DL2, 2DL3, 2DL4, 2DL5, 3DL1, 3DL2, 3DL3, 2DS1, 2DS2, 2DS3, 2DS5 and 3DS1) in the above-mentioned 62 populations, we drew a MDS plot (stress 0.0119) which showed an excellent configuration quality in Fig. 5. The dimensionality reduction played a crucial role in showing the relationship among our studied population and 61 other reported populations in the graph. The Eastern Han, Jilin Han and Shanghai Han clung in the low left quadrant together with Yunnan Han, Shenzhen Han, and Singapore Han. African populations were close to East Asian groups while American and European groups were relatively near to Central and West Asian groups. This is particularly interesting and provides some potential evidences to the human population migration. The close relationship between Eastern Han and Jilin Han exactly corresponded with the findings by phylogenetic reconstruction. Compared with various African, European, and American populations, the Han populations were all closely located in negative axis of dimension 1. The results of MDS analysis strongly supported the findings from population evolution researches on 62 populations in Neighbor-Joining tree.

### Population differentiation of Han ethnic group in East Asia

As KIR genomic content varied from population to population^63^, the changes in various subgroups of a certain ethnic group would reveal the impact of migration, environment and marriage on population differentiation. Our analysis on population genetics (the Heatmap, NJ tree, and MDS plot) about Eastern Chinese Han revealed that this population was close to various mainland Han groups in China in different degree. Han ethnic group, the largest population in China with the amount of 1,225,932,641, accounting for 91.51% in the 6^th^ nationwide census, requires comprehensive and intensive researches on population genetics. Based on the carrier frequencies of Eastern Han group and previously published Han populations including Shanghai Han, Jilin Han, Xinjiang Han, Yunnan Han, Sichuan Han, Shenzhen Han, Shaanxi Han, Hong Kong Han and Singapore Han, we conducted a chi-square (X^2^) test to depict KIR loci with significant differences. In Table 5, we listed 17 comparisons of KIR loci among 10 Han populations distributed around China and Singapore. Irrespective of all framework genes or pseudogenes, we found that 6 KIR genes (KIR3DL1, 2DL1, 2DS4, 2DS1, 2DS5 and 1D, p < 0.0005) were determined to be highly polymorphic KIR loci, indicating that these loci might be useful in the studies of forensic and population genetics. As described in IPD-KIR database (http://www.ebi.ac.uk/ipd/kir/stats.html), the number of alleles and encoded proteins varied among 16 KIR genes. The most polymorphic KIR gene is 3DL2 with 112 alleles and 82 proteins, followed by 3DL3 and 3DL1. Our observation of 6 remarkably polymorphic KIR loci among 10 Han populations was inconsistent with the IPD-KIR database. The IPD-KIR database points the diversities of KIRs in terms of the variants in genomes and proteomes while our research observed the gene diversities by changes in OFs of KIRs among various Han populations. After X^2^ analysis, 3DL1, 2DL1, 2DS4, 2DS1, 2DS5, and 1D were figured out to be highly polymorphic KIR loci among 10 Han populations. Though some KIRs have been proved to be highly polymorphic in IPD-KIR database, such as 2DL2 and 2DL3, they are less variable in Chinese Han populations. The possible cause of the inconformity is probably the heterogeneities of KIRs in different populations. Another prevalent puzzle concerning KIR diversity was the underlying mechanism by which KIR gene content polymorphisms and interactions between KIR and HLA molecules influence various immune-related diseases, which was still ambiguous^11^,^64^,^65^,^66^. This study not only provided effective genetic markers for forensic and population genetics, but also benefited in exploring the susceptible KIR genes in immune diseases, this will require comprehensive analysis of samples from both health and disease conditions.

## Concluding Remarks

People, from different geographical regions and different ethnic groups, possessed KIR genotypes with high variability. Hence, we detected polymorphisms of KIR genes from Han population living in eastern coastal area of China (Jiangsu province) using PCR-SSP method. The purpose of present study was to provide the genetic information on KIRs distribution in Eastern Chinese Han population. We successfully identified the genotype of 17 KIR genes in 123 unrelated healthy donors in Eastern Chinese Han. The OFs (accordingly GFs) ranged from 14.63% (7.60%) in KIR2DS3 to 100% (100%) in KIR2DL3, 2DL1, and 2DP1. As well, the 4 framework genes were existed in all individuals. The OFs of A group genes were all greater than B group. The activating genes KIR2DS4 occurred more frequently while inhibitory genes KIR2DL2 and 2DL5 showed lower OF, it is consistent to previous report^18^,^19^,^22^,^23^. A total of 27 distinct genotypes were identified. The most common genotype was KIR 3DL1-2DL1-2DL3-2DS4-2DL4-3DL2-3DL3-2DP1-3DP1 (n = 63, ratio = 51.22%). A total of 7 BB genotypes, 53 AB genotypes and 63 AA genotypes were determined. As for linkage group, among 60 samples categorized as Bx haplogroup, 30 were from CxTx, 23 were from CxT4, 6 were from C4Tx and 1 were from C4T4. We conducted LD analysis using D as the test parameter. The 45 pairwise comparisons of 10 genes were performed with the exclusion of 7 genes detected in all individuals, and we found 35 strong relations, among which 6 associations were negative, all related with KIR1D. The data of this study not only reiterated what we already know about the Asian/Han Chinese populations, but also provided a clear KIR gene genotype distribution and linkage pattern of KIR pairs in Eastern Chinese Han population.

As for clustering analysis and phylogenetic reconstruction, we utilized Heatmap, Neighbor-Joining tree and MDS plot analysis. The data from Heatmap showed that Eastern Han had close relationship with Han from HGDP-CEPH, Xinjiang Han, Chinese Dai, Yunnan Han, Eastern Chinese Han, Shenzhen Han, Shaanxi Han and Jilin Han. Next, we employed Dispan to generate the D_A_ distances by the carrier frequencies of 13 KIR loci in 62 populations. Then, the D_A_ distances and illustrated NJ-tree indicated a close relationship between Eastern Han and Jilin Han from Northeastern China, as well as Eastern Han and Shanghai Han, and the result was strongly supported by the MDS plot analysis. X^2^ analysis was also performed and we found that KIR3DL1, 2DL1, 2DS4, 2DS1, 2DS5 and 1D are highly polymorphic KIR loci specifically to Han Chinese. In conclusion, this research on Eastern Han characterized by generating KIR genotyping files, phylogenetic construction, evolutionary molecular analysis and sub-Han group comparison provided valuable resources for both enriching information pool of forensic and population genetics and benefiting in uncovering underlying genetic mechanisms underlying immune diseases in the future. The comprehensive and comparative analysis on KIRs revealed a unique genetic background of Eastern Chinese Han through phylogenetic construction, evolutionary molecular analysis, and sub-Han group comparison which provided valuable resources for both enriching information pool of forensic and population genetics.

## Additional Information

**How to cite this article**: Yin, C. *et al*. Genetic polymorphism and evolutionary differentiation of Eastern Chinese Han: a comprehensive and comparative analysis on KIRs. *Sci. Rep.* **7**, 42486; doi: 10.1038/srep42486 (2017).

**Publisher's note:** Springer Nature remains neutral with regard to jurisdictional claims in published maps and institutional affiliations.

## Supplementary Material

Supplementary Information

Supplementary Dataset 1

## Figures and Tables

**Table 1:** The OFs and estimated GFs of KIR cluster genes in the Eastern Chinese Han.

Frequencies	Inhibitory KIR								Pseudogenes		Activating KIR						
	KIR	KIR	KIR	KIR	KIR	KIR	KIR	KIR	KIR	KIR	KIR	KIR2	KIR	KIR	1D	KIR	KIR
	2DL1	2DL2	2DL3	2DL4	2DL5	3DL1	3DL2	3DL3	2DP1	3DP1	2DS1	DS2	2DS3	2DS4		2DS5	3DS1
OF	1.0000	0.2033	1.0000	1.0000	0.3577	0.9593	1.0000	1.0000	1.0000	1.0000	0.3659	0.1789	0.1463	0.9431	0.4309	0.2927	0.3008
GF	1.0000	0.1074	1.0000	1.0000	0.1986	0.7983	1.0000	1.0000	1.0000	1.0000	0.2037	0.0939	0.0760	0.7615	0.2456	0.1590	0.1638

OF: observed carrier frequency

GF: gene frequency

**Table 2:** Loci genotyping profiles observed on Killer cell immunoglobulin-like receptors (KIRs) in the Eastern Chinese Han(n = 123).

Haplo group	Geno type	N	KIR 3DL1	KIR 2DL1	KIR 2DL3	KIR 2DS4	KIR 2DL2	KIR 2DL5	KIR 3DS1	KIR 2DS1	KIR 2DS2	KIR 2DS3	KIR 2DS5	KIR 2DL4	KIR 3DL2	KIR 3DL3	KIR 2DP1	KIR 3DP1	ID	Genotype	Linkage Group
AA	1	31	+	+	+	+								+	+	+	+	+		AA	CxTx
AA	1	32	+	+	+	+								+	+	+	+	+	+	AA	CxTx
Bx	2	11	+	+	+	+		+	+	+			+	+	+	+	+	+		AB	CxT4
Bx	2	2	+	+	+	+		+	+	+			+	+	+	+	+	+	+	AB	CxT4
Bx	3	2	+	+	+	+	+	+	+	+	+		+	+	+	+	+	+		AB	CxT4
Bx	3	3	+	+	+	+	+	+	+	+	+		+	+	+	+	+	+	+	AB	CxT4
Bx	4	2	+	+	+	+	+				+			+	+	+	+	+		AB	CxTx
Bx	4	3	+	+	+	+	+				+			+	+	+	+	+	+	AB	CxTx
Bx	5	3	+	+	+	+	+	+			+	+		+	+	+	+	+		AB	C4Tx
Bx	7	2	+	+	+	+	+	+	+	+	+	+		+	+	+	+	+	+	AB	C4Tx
Bx	8	6	+	+	+	+		+	+	+		+		+	+	+	+	+		AB	CxTx
Bx	8	1	+	+	+	+		+	+	+		+		+	+	+	+	+	+	AB	CxTx
Bx	9	1	+	+	+	+	+	+		+	+		+	+	+	+	+	+	+	AB	CxTx
Bx	11	1	+	+	+	+	+	+		+	+	+		+	+	+	+	+		AB	C4Tx
Bx	19	1	+	+	+	+	+							+	+	+	+	+		AB	CxTx
Bx	19	1	+	+	+	+	+							+	+	+	+	+	+	AB	CxTx
Bx	23	1	+	+	+	+							+	+	+	+	+	+	+	AB	CxTx
Bx	31	1	+	+	+	+	+	+			+			+	+	+	+	+		AB	CxTx
Bx	32	1	+	+	+	+		+					+	+	+	+	+	+	+	AB	CxTx
Bx	35	2	+	+	+	+		+		+			+	+	+	+	+	+	+	AB	CxTx
Bx	44	1	+	+	+	+	+				+		+	+	+	+	+	+	+	AB	CxTx
Bx	68	1		+	+		+	+	+	+	+		+	+	+	+	+	+		BB	CxT4
Bx	69	1		+	+			+	+	+			+	+	+	+	+	+		BB	CxT4
Bx	70	1		+	+		+	+	+	+	+	+	+	+	+	+	+	+		BB	C4T4
Bx	75	2		+	+			+	+	+		+	+	+	+	+	+	+		BB	CxT4
Bx	79	1	+	+	+			+	+	+			+	+	+	+	+	+		BB	CxT4
Bx	200	1	+	+	+	+		+						+	+	+	+	+		AB	CxTx
Bx	202	2	+	+	+	+			+	+			+	+	+	+	+	+	+	AB	CxTx
Bx	331	1	+	+	+			+	+	+		+		+	+	+	+	+		BB	CxTx
Bx	370	1	+	+	+	+	+			+	+		+	+	+	+	+	+		AB	CxTx
Bx	371	1	+	+	+	+	+			+			+	+	+	+	+	+	+	AB	CxTx
Bx	372	1	+	+	+	+			+	+		+		+	+	+	+	+		AB	CxTx
Bx	439	2	+	+	+	+				+			+	+	+	+	+	+		AB	CxTx

Cells filled with “+” symbol and blank cells represent presence and absence respectively.

**Table 3:** Linkage disequilibrium coefficients of 45 KIR comparison pairs in Eastern Chinese Han.

Genes		2DS4	1D	2DL2	2DL5	3DS1	2DS1	2DS2	2DS5	2DS3
3DL1	D	0.0383	−0.0174	0.0080	0.0260	0.0283	0.0257	0.0090	0.0287	0.0184
	p	**0.0000**	**0.0052**	0.1138	**0.0000**	**0.0000**	**0.0000**	0.0621	**0.0000**	**0.0000**
2DS4	D		−0.0245	0.0047	0.0365	0.0397	0.0360	0.0061	0.0321	0.0242
	p		**0.0008**	0.4297	**0.0000**	**0.0000**	**0.0000**	0.2825	**0.0000**	**0.0000**
1D	D			0.0100	−0.0566	−0.0483	−0.0438	0.0042	−0.0123	−0.0387
	p			0.4323	**0.0002**	**0.0008**	**0.0040**	0.7270	0.3915	**0.0005**
2DL2	D				0.0492	0.0120	0.0313	0.1424	0.0299	0.0272
	p				**0.0001**	0.3069	**0.0113**	**0.0000**	**0.0103**	**0.0028**
2DL5	D					0.1688	0.1781	0.0580	0.1229	0.0859
	p					**0.0000**	**0.0000**	**0.0000**	**0.0000**	**0.0000**
3DS1	D						0.1907	0.0194	0.1233	0.0698
	p						**0.0000**	0.0839	**0.0000**	**0.0000**
2DS1	D							0.0321	0.1612	0.0684
	p							**0.0064**	**0.0000**	**0.0000**
2DS2	D								0.0290	0.0308
	p								**0.0092**	**0.0004**
2DS5	D									−0.0185
	p									0.0719

D: LD coefficient; Statistically significant P values (p < 0.05) are labeled bold and underlined.

**Table 4:** D_A_ and p values among Eastern Chinese Han and 21 representative populations.

	Eastern Han	Bantu S	Mbuti Pygmies	San	Palestinian	Adygei	French	Russian	Hazara	Balochi	Barusho	Uygur	Daur	CEPH-Han	Japanese	Mongola	Melanesian	Maya	Colombian	Shanghai Han	Jilin Han	HongKong Han
EasternHan	*	0.0104	0.0537	0.1043	0.0401	0.0453	0.0175	0.0023	0.0429	0.0248	0.0507	0.006	0.006	0.0029	0.0031	0.0049	0.1145	0.0151	0.0368	0.0004	0.0002	0.0011
Bantu S	0.0272	*	0.0699	0.0894	0.0322	0.056	0.0236	0.0061	0.0542	0.0364	0.0501	0.0198	0.0057	0.0195	0.0187	0.0244	0.1274	0.0457	0.073	0.0125	0.0105	0.0156
Mbuti Pygmies	**0.076**	**0.0621**	*	0.021	0.0471	0.0412	0.037	0.0513	0.0369	0.0479	0.0312	0.0293	0.087	0.0557	0.0593	0.0265	0.1259	0.0373	0.0503	0.0584	0.0579	0.0511
San	**0.1493**	**0.1123**	0.0453	*	0.0286	0.0475	0.0436	0.0692	0.0417	0.0498	0.0307	0.0626	0.1445	0.0833	0.1476	0.0677	0.1078	0.0644	0.0656	0.1058	0.1088	0.0836
Palestinian	0.042	0.0502	0.0572	0.0714	*	0.0093	0.008	0.0228	0.0092	0.0044	0.0066	0.0167	0.0611	0.0225	0.0627	0.0373	0.0349	0.0235	0.0399	0.0401	0.0431	0.0254
Adygei	0.0497	**0.0744**	0.0586	0.0809	0.0107	*	0.0099	0.0317	0.0029	0.0061	0.0041	0.0162	0.0773	0.029	0.0687	0.0337	0.0283	0.0198	0.0278	0.0459	0.0487	0.0305
French	0.0197	0.038	0.058	0.093	0.0111	0.0103	*	0.0073	0.0142	0.0022	0.0111	0.0057	0.0326	0.0091	0.0355	0.0188	0.051	0.0094	0.0226	0.0181	0.0194	0.0093
Russian	0.0115	0.016	**0.0539**	**0.1048**	0.0263	0.0384	0.0127	*	0.0317	0.0149	0.0326	0.0045	0.0088	0.0053	0.0108	0.0081	0.0993	0.0207	0.0402	0.0024	0.0027	0.0026
Hazara	0.0518	**0.0713**	0.0506	0.0736	0.0093	0.0021	0.0131	0.036	*	0.0075	0.0022	0.0141	0.0754	0.0261	0.0635	0.0267	0.0336	0.0142	0.0203	0.0431	0.046	0.0285
Balochi	0.0253	0.0578	0.0795	0.1117	0.0113	0.0135	0.0084	0.0285	0.0189	*	0.0069	0.0091	0.046	0.0121	0.0447	0.0249	0.0318	0.0136	0.0275	0.0249	0.0271	0.014
Barusho	**0.0587**	**0.073**	0.0554	0.0664	0.0089	0.0101	0.0197	0.0367	0.0074	0.021	*	0.021	0.0812	0.0329	0.0788	0.0346	0.0307	0.0209	0.0252	0.0515	0.0541	0.035
Uygur	0.0088	0.0435	0.0629	**0.1207**	0.0216	0.0241	0.0101	0.0171	0.0274	0.0076	0.0324	*	0.017	0.0053	0.0162	0.0075	0.071	0.0032	0.0115	0.0058	0.0066	0.0036
Daur	0.0191	0.026	**0.1118**	**0.1954**	**0.0913**	**0.1091**	0.0593	0.0299	**0.1101**	0.0769	**0.1191**	0.0471	*	0.0157	0.0106	0.0181	0.1721	0.0414	0.0761	0.006	0.0049	0.0099
CEPH|-Han	0.0047	0.0325	**0.0782**	**0.1384**	0.025	0.0309	0.0109	0.0127	0.0336	0.0111	0.0403	0.0049	0.035	*	0.009	0.012	0.0711	0.0139	0.0326	0.0027	0.0038	0.0008
Japanese	0.0073	0.0474	**0.1017**	**0.1959**	**0.0669**	**0.075**	0.0425	0.0285	**0.0759**	0.0453	**0.0851**	0.021	0.0206	0.0159	*	0.0127	0.1398	0.0197	0.0462	0.0027	0.0025	0.0073
Mongola	0.0129	0.0577	0.0641	**0.1367**	0.0468	0.0463	0.0276	0.0258	0.0472	0.0284	0.054	0.0097	0.0483	0.0176	0.0213	*	0.1129	0.0031	0.0105	0.0064	0.0058	0.0082
Melanesian	**0.1567**	**0.1854**	**0.1554**	**0.1456**	0.0558	0.0598	**0.0973**	**0.1394**	0.0578	0.0713	0.0474	**0.1063**	**0.2472**	**0.117**	**0.1783**	**0.1462**	*	0.0804	0.098	0.1134	0.1208	0.0834
Maya	0.02	0.0608	0.0564	**0.1161**	0.0243	0.0187	0.0132	0.0201	0.0185	0.0154	0.0219	0.0079	0.0681	0.0147	0.0313	0.0121	**0.0952**	*	0.0025	0.016	0.017	0.0118
Colombian	0.0454	**0.1065**	**0.0676**	**0.1275**	0.0494	0.0329	0.0372	0.0555	0.0344	0.0323	0.0443	0.0237	**0.1081**	0.0399	0.0577	0.0152	**0.1098**	0.0136	*	0.039	0.0404	0.0305
Shanghai Han	0.0036	0.0242	**0.0739**	**0.1446**	0.0408	0.0476	0.0181	0.0053	0.0474	0.031	**0.0535**	0.0145	0.0186	0.0076	0.0119	0.019	**0.1576**	0.0193	0.0514	*	0.0005	0.001
Jilin Han	0.0006	0.0265	**0.0771**	**0.1509**	0.043	0.0538	0.0224	0.0101	0.0547	0.0281	**0.0588**	0.0104	0.0179	0.0062	0.0066	0.0135	**0.1574**	0.0204	0.0481	0.003	*	0.0016
HongKong Han	0.0035	0.0276	**0.0698**	**0.1302**	0.024	0.0335	0.0109	0.0069	0.0343	0.0142	0.0368	0.0049	0.0308	0.0019	0.0141	0.0152	**0.12**	0.0121	0.0405	0.0045	0.0033	*

Statistically no significant difference (p > 0.05) are indicated in underlined format and according D_A_ distances are indicated in bold.

**Table 5:** The chi-square (X^2^) results of various KIR loci in 10 Han populations.

Populations	Number	Framework genes or pseudogene				Functional genes													Citations
		3DL3	2DL4	3DL2	3DP1	3DL1	2DL1	2DL3	2DS4	2DL2	2DL5	3DS1	2DS1	2DS2	2DS3	2DS5	2DP1	1D	
Eastern Han	123	1.0000	1.0000	1.0000	1.0000	0.9593	1.0000	1.0000	0.9431	0.2033	0.3577	0.3008	0.3659	0.1789	0.1463	0.2927	1.0000	0.4309	Current Study
Shanghai Han	87	1.0000	1.0000	1.0000	—	1.0000	0.9770	0.9770	0.7586	0.1839	0.3793	0.3448	0.3563	0.1839	0.1494	0.2414	—	0.4713	25
Jilin Han	201	1.0000	1.0000	1.0000	0.9950	0.9403	0.9950	0.9950	0.7512	0.1791	0.3483	0.3035	0.3433	0.1692	0.1244	0.2637	0.9900	—	27
Xinjiang Han	184	1.0000	1.0000	1.0000	1.0000	0.9130	0.9946	0.9891	0.8913	0.2228	0.4837	0.4402	0.4565	0.2174	0.2391	0.3207	0.9946	—	30
Yunnan Han	404	1.0000	1.0000	1.0000	0.9950	0.9554	0.9950	0.9876	0.9579	0.1807	0.3688	0.3886	0.3540	0.1807	0.1658	0.3020	0.9950	—	22
Sichuan Han	286	1.0000	1.0000	1.0000	0.9930	0.9650	0.9860	0.9755	0.8916	0.2483	0.4266	0.3566	0.7273	0.2413	0.1958	0.2657	0.9860	0.3357	29
Shenzhen Han	503	1.0000	1.0000	1.0000	1.0000	0.9620	0.9920	0.9840	0.9620	0.2070	0.3760	0.3360	0.3260	0.2070	0.1850	0.2290	0.9920	—	28
Shaanxi Han	104	1.0000	1.0000	1.0000	1.0000	0.9600	0.9400	0.9800	0.6900	0.1900	0.4100	0.3900	0.3900	0.2100	0.1400	0.3800	1.0000	0.3300	18
Hong Kong Han	46	1.0000	1.0000	1.0000	—	0.9400	0.9900	0.9800	—	0.2800	0.4500	0.3900	0.4000	0.2800	0.2500	0.2600	—	—	19
Singapore Han	100	1.0000	1.0000	1.0000	—	0.9800	1.0000	1.0000	—	0.2800	0.3900	0.3000	0.2800	0.2800	0.1700	0.2200	—	—	19
chi-square test	P-value	NS(1)	NS(1)	NS(1)	0.3020	**0.0290**	**0.0160**	0.2860	**0.0000**	0.2960	0.1840	0.0970	**0.0000**	0.2690	0.1200	**0.0440**	0.4980	**0.0480**	

Significant differences (p < 0.05) were labeled with bold and underlined format.
