# Supplementary material for: Genetic polymorphism and evolutionary differentiation of Eastern Chinese Han: a comprehensive and comparative analysis on KIRs
Source: Sci Rep. 2017 Feb 16;7:42486. doi: 10.1038/srep42486 (PMC5311978; doi:10.1038/srep42486)
Supplement: Supplementary Information [file srep42486-s1.pdf]

Supplementary Information

Title: Genetic polymorphism and evolutionary differentiation of Eastern Chinese Han: a comprehensive and comparative analysis on KIRs

Caiyong Yin[1], Li Hu[1], Huijie Huang[1], Yanfang Yu[1], Zheng Li[1], Qiang Ji[1], Xiaochao Kong[1], Zhongqun Wang[5], Jinchuan Yan[5], Jiangwei Yan[6], Bofeng Zhu[2,3,4], Feng Chen[1]

[1]Department of Forensic Medicine, Nanjing Medical University, Nanjing, Jiangsu, 210029, China

[2]Department of Forensic Genetics, School of Forensic Medicine, Southern Medical University, Guangzhou 510515, P. R. China

[3]Key Laboratory of Shaanxi Province for Craniofacial Precision Medicine Research, College of Stomatology, Xi'an Jiaotong University, Xi'an, Shaanxi 710004, P. R. China

[4]Clinical Research Center of Shaanxi Province for Dental and Maxillofacial Diseases, College of Stomatology, Xi'an Jiaotong University, Xi'an, Shaanxi 710004, P. R. China

[5]Department of Cardiology, Affiliated Hospital of Jiangsu University, Zhenjiang, Jiangsu 212001, China.

[6]CAS Key Laboratory of Genome Sciences and Information, Beijing Institute of Genomics, Chinese Academy of Sciences, Beijing, China.

*Correspondence should be addressed to Feng Chen, Department of Forensic Medicine, Nanjing Medical University, China, Email: fchen@njmu.edu.cn

The supplementary file contains Figure S1 and its legend.

Figure S1

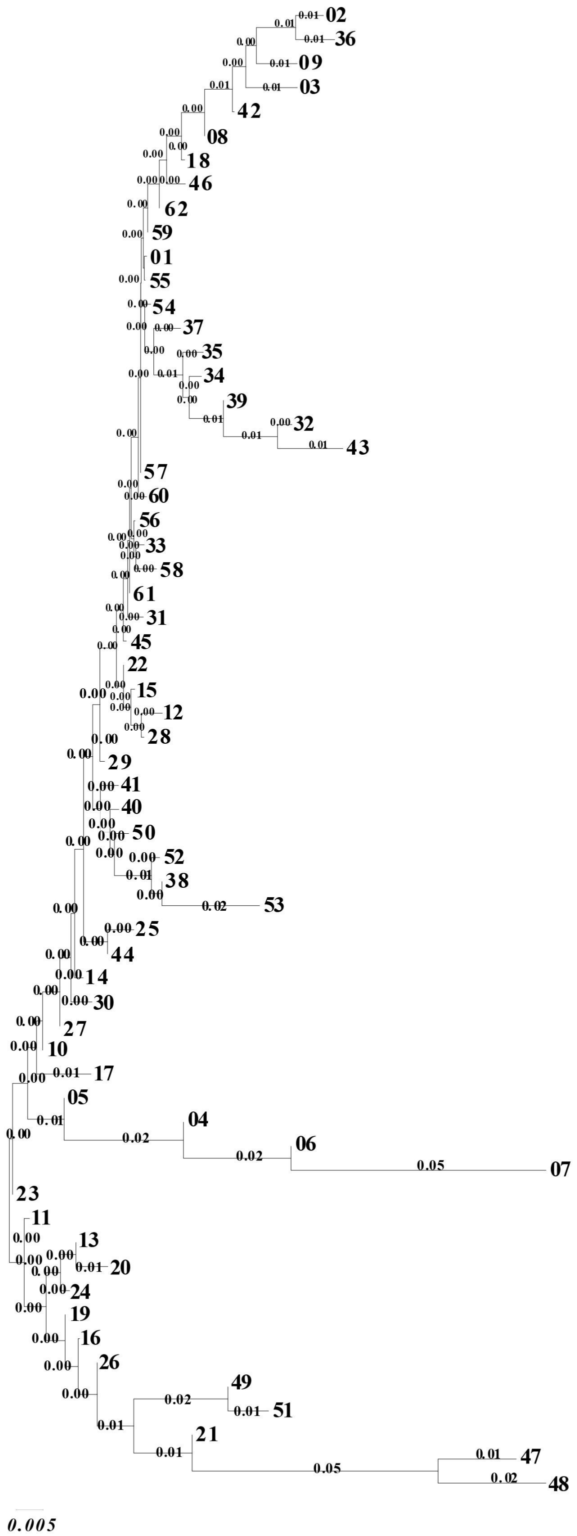

Figure S1. The interior branch test was conducted to assess the reliability of N-J phylogenetic reconstruction. The interior branch length and the ruler were marked accordingly (the sum of branch length = 0.41784153).
